# Single-cell transcriptome analysis identifies a novel tumor-associated macrophage subtype predicting better prognosis in pancreatic ductal adenocarcinoma

**DOI:** 10.3389/fcell.2024.1466767

**Published:** 2024-10-23

**Authors:** Xiaonan Wang, Dongyi Li, Bo Zhu, Zichun Hua

**Affiliations:** ^1^ School of Biopharmacy, China Pharmaceutical University, Nanjing, China; ^2^ The State Key Laboratory of Pharmaceutical Biotechnology, School of Life Sciences, Nanjing University, Nanjing, China; ^3^ Faculty of Pharmaceutical Sciences, Xinxiang Medical University, Xinxiang, China; ^4^ Changzhou High-Tech Research Institute, Nanjing University, Changzhou, China

**Keywords:** single-cell sequencing, pancreatic cancer, tumor associated macrophages, tumor microenvironment, prognosis

## Abstract

**Background:**

Characterized by an immune-suppressive tumor microenvironment (TME), pancreatic ductal adenocarcinoma (PDAC) is well-known for its poor prognosis. Tumor associated macrophages (TAMs) play a critical role in PDAC TME. An in-depth understanding of TAMs is helpful to develop new strategies for immunotherapy.

**Methods:**

A large number of single-cell RNA sequencing data and bulk RNA sequencing data of PDAC were collected for systematic bioinformatics analysis. Characterize subtypes of TAMs at single-cell resolution and its effect on prognosis. Differential gene analysis and cell-cell communication were used to describe the effect on prognosis and validated by the TCGA dataset.

**Results:**

We used two prognosis-favorable genes, SLC12A5 and ENPP2, to identify a benign M2-like TAMs (bM2-like TAMs), which shared similarities with C1QC + TAMs, CXCL9+ TAMs and CD169+ TAMs, by analyzing scRNA-seq data and bulk RNA data of PDAC. The bM2-like TAMs were revealed to promote T cell activation and proliferation through ALCAM/CD6 interaction. Meanwhile, the bM2-like TAMs were responsible for stroma modeling by altering αSMA+/αSMA-cell ratio. On the contrast, the rest of the M2-like TAMs were defined as malignant M2-like TAMs (mM2-like TAMs), partly overlapping with SPP1+ TAMs. mM2-like TAMs were revealed to promote tumor progression by secretion of MIF and SPP1.

**Conclusion:**

Our study used two prognosis-favorable genes to divide M2-like TAMs of PDAC into anti-tumor bM2-like TAMs and pro-tumor mM2-like TAMs. The bM2-like TAMs activate T cells through ALCAM/CD6 and generate prognosis-favorable αSMA+ myofibroblasts through secreting TGFβ, which brings insight into heterogeneity of TAMs, prognosis prediction and immunotherapy of PDAC.

## 1 Introduction

Pancreatic ductal adenocarcinoma (PDAC) is the most prevalent pancreatic tumor, accounting for 90% of all pancreatic malignancies (Siegel et al., 2023). PDAC is one of the deadliest cancers and is known as the “king of cancer” due to its poor prognosis, short median survival and high mortality rate. Due to the special anatomical location of the pancreas, early symptoms of PDAC are not obvious, and most patients are diagnosed in the progressive or metastatic stage (Takikawa et al., 2022). In addition, less than 20 percent of patients are eligible for surgery (Kleeff et al., 2016; Ryan et al., 2014). Unfortunately, even after surgical resection, approximately 50%–70% of patients will relapse within 1 year, with a 5-year survival rate of only 8%–10% (Groot et al., 2018). Systemic chemotherapy remains the dominant therapy. One is FOLFIRINOX, a combination of 5-fluorouracil (5-FU), leucovorin, irinotecan and oxaliplatin (Conroy et al., 2011). The other is a combination of gemcitabine and albumin-paclitaxel (NAB-paclitaxel) (Von Hoff et al., 2013). Overall, there are no universal standards for second-line regimens and beyond in PDAC, and the course of therapy is often determined by the patient’s performance status, presence of “actionable” targets, and the availability of appropriate clinical trials. An in-depth exploration of the mechanisms underlying the poor prognosis of PDAC from a novel perspective may provide new insights into the development of potential therapeutic strategies.

The tumor microenvironment (TME) is complex and composed of a large number of fibroblasts, a dense extracellular matrix, a disordered vascular system, and various immune cells (Halbrook et al., 2023). The resulting interstitial density provides a cozy niche for tumor cell proliferation and a physical barrier to the penetration of drugs and anti-tumor immune cells (Hegde et al., 2020). The TME in PDAC is highly immunosuppressed. Fibrous tissue constitutes 80% of the volume of PDAC tissue and plays a key role in tumor development. Myeloid cells are the predominant immune cell population in pancreatic cancer tissue, constituting approximately 40% of the total immune cells (Guo et al., 2023). Myeloid cells, such as tumor-associated macrophages (TAMs), play a key role in the initial and progression of PDAC. With the complex and heterogeneous composition of myeloid cells, it is biologically and clinically important to further subdivide and characterize new cell subtypes based on characteristic gene expression status to clarify their prognostic functions. In particular, exploring the communication of myeloid cells with other components of the TME contributes to a deeper understanding of myeloid cell function (Xiong et al., 2019; Liu X. et al., 2022).

Developments in single-cell genomics have provided powerful tools for exploring genetic and functional heterogeneity, mapping immune profiles, and detecting rare subpopulations (Giladi and Amit, 2018; Yofe et al., 2020). Single-cell RNA sequencing (scRNA-seq) can effectively characterize the high heterogeneity of tumors and reveal the dynamic changes in cancer occurrence and metastasis driven by the genetic and immune microenvironment (González-Silva et al., 2020). However, most studies using the scRNA-seq technique have a small number of samples and the interpretation of heterogeneity is somewhat narrow. In this study, we explored the subtypes of immune cells, especially TAMs, in the TME based on single-cell sequencing data from a relatively large sample of human PDAC. The aim of this study is to provide new perspectives for immunotherapy of PDAC through an in-depth and detailed analysis of myeloid cell fractions in TME.

## 2 Materials and methods

### 2.1 Data source

Single-cell sequencing data included in this study were obtained from the Gene Expression Omnibus (GEO) database (https://www.ncbi.nlm.nih.gov/geo/). The dataset consists of 27 primary tumor samples (Accession numbers: GSE154778 and GSE155698), 10 metastatic tumor samples (Accession numbers: GSE156405 and GSE154778), and 3 adjacent normal samples (Accession number: GSE155698) (see Table 1 for details). In addition, bulk RNA-seq data from 172 pancreatic adenocarcinoma (PAAD) patients with clinical information were acquired from The Cancer Genome Atlas (TCGA) database (https://www.cancer.gov/ccg/research/genome-sequencing/tcga).

**TABLE 1:** Details of scRNA-seq datasets.

GEO series	GEO sample	Characteristics	bTAMs	Samples
GSE154778	GSM4679532	primary tumor	bTAMs_medium	10
	GSM4679533	primary tumor	bTAMs_medium	
	GSM4679534	primary tumor	bTAMs_high	
	GSM4679535	primary tumor	bTAMs_low	
	GSM4679536	primary tumor	bTAMs_high	
	GSM4679537	primary tumor	bTAMs_high	
	GSM4679538	primary tumor	bTAMs_high	
	GSM4679533	primary tumor	bTAMs_high	
	GSM4679540	primary tumor	bTAMs_high	
	GSM4679541	primary tumor	bTAMs_high	
GSE155698	GSM4710689	primary tumor	bTAMs_medium	15
	GSM4710690	primary tumor	bTAMs_low	
	GSM4710691	primary tumor	bTAMs_low	
	GSM4710692	primary tumor	bTAMs_low	
	GSM4710693	primary tumor	bTAMs_medium	
	GSM4710694	primary tumor	bTAMs_medium	
	GSM4710695	primary tumor	bTAMs_high	
	GSM4710696	primary tumor	bTAMs_high	
	GSM4710697	primary tumor	bTAMs_low	
	GSM4710698	primary tumor	bTAMs_low	
	GSM4710699	primary tumor	bTAMs_medium	
	GSM4710700	primary tumor	bTAMs_low	
	GSM4710701	primary tumor	bTAMs_low	
	GSM4710702	primary tumor	bTAMs_medium	
	GSM4710703	primary tumor	bTAMs_low	
	GSM4710704	primary tumor	bTAMs_high	
	GSM4710705	primary tumor	bTAMs_high	
GSE156405	GSM4730265	Vaginal metastasis	bTAMs_medium	4
	GSM4730266	Liver metastasis	bTAMs_medium	
	GSM4730267	Lung metastasis	bTAMs_high	
	GSM4730268	Peritoneal metastasis	bTAMs_medium	
GSE154778	GSM4679542	Liver metastasis	bTAMs_medium	6
	GSM4679543	Liver metastasis	bTAMs_low	
	GSM4679544	Omentum metastasis	bTAMs_low	
	GSM4679545	Liver metastasis	bTAMs_low	
	GSM4679546	Liver metastasis	bTAMs_medium	
	GSM4679547	Liver metastasis	bTAMs_medium	
GSE155698	GSM4710706	Adjacent normal tissue	bTAMs_high	3
	GSM4710707	Adjacent normal tissue	bTAMs_low	
	GSM4710708	Adjacent normal tissue	bTAMs_low	

### 2.2 Data preprocessing

R studio version 4.1.2 was used for the analysis of scRNA-seq. Read-in, quality control and preprocessing of scRNA-seq data were performed using the R package Seurat version 4.1.0 (https://satijalab.org/seurat/) (Hao et al., 2021). During quality control, cells considered unqualified were defined based on the following criteria: 1) Cells with fewer than 200 genes detected; 2) Cells with more than 7,500 genes detected; 3) Cells with more than 20% mitochondrial-specific genes detected (Zhao et al., 2023; Nalio Ramos et al., 2022; Bian et al., 2020). Unqualified cells and genes that were detected in fewer than 3 cells were removed from datasets in subsequent analyses. A total of 80,273 cells remained for further analyses.

Gene expression levels were normalized (LogNormalize) with “NormalizedData” function and the 2000 most variable genes were identified (Chen et al., 2022). The normalized matrix was then scaled before principal component analysis (PCA) was performed. To remove the batch effect, the R package Harmony version 0.1.0 (Korsunsky et al., 2019) was used, after which the top 50 harmony dimensions were selected for nonlinear dimension reduction and clustering. Uniform manifold approximation and projection (UAMP) and t-DistributedStochastic Neighbor Embedding (t-SNE) were used to group and visualize all cells. Cell types were annotated according to previous reports (Elyada et al., 2019; Wang et al., 2021) based on the clustering result.

### 2.3 Single-cell copy number variation (CNV) evaluation

Single-cell datasets were used for copy number variation analysis to show how malignant the epithelial cells were. The CNV evaluation of each cell was performed using infercnv R package (version 1.4.0; https://github.com/broadinstitute/inferCNV/wiki) (Patel et al., 2014). InferCNV can provide evidence of large-scale chromosomal copy number alterations in cells, such as the increase or loss of whole chromosomes or large chromosomal segments. Chromosomal variation can be inferred by exploring the intensity of gene expression at different locations in the tumor genome compared to normal cells. The inferCNV analysis was performed with parameters including “denoise”, default settings for the Hidden Markov model (HMM), and a value of 0.1 for “cutoff”. To reduce false positive CNV calls, 500 immune cells and 500 stromal cells were included in the analysis as controls.

### 2.4 Pseudotime trajectory analysis

Single-cell datasets were used for pseudotime trajectory analysis to characterize the differentiation pattern of the TAMs. Specifically, two clusters of TAMs with a total of 11,962 cells were separated and pseudotime trajectory analysis was performed using the R package Monocle version 2.22.0. TAMs from 40 samples were placed on the pseudotime trajectory to identify possible differentiation patterns of TAMs under physiological conditions. State 5 was considered as the root state for cell ordering according to the already known different pattern of TAMs.

### 2.5 Kyoto encyclopedia of genes and genomes (KEGG) analysis

KEGG analyzed the differential expression genes of TAM in the single-cell datasets and quickly screened the pathway where the differential genes were located. Using R package clusterProfiler version 4.2.2 (Wu et al., 2021), KEGG analysis was performed on the differentially expressed genes (DEGs) identified between the subclusters of bM2-like TAMs and mM2-like TAMs with adjusted *p-value* less than 0.05 and *|*log*2FC|* more than 0.2. Genome wide annotation R package org. Hs.eg.db version 3.14.0 was used for ID transition. In the KEGG analysis, “BH” method was used to adjust the *p-value.*

### 2.6 Gene set enrichment analysis (GSEA)

GSEA was used to further analyze the influence of differential genes on specific pathways in single-cell datasets. GSEA does not need to specify an explicit differential gene threshold, and the algorithm analyzes differences based on actual overall trends. All DEGs (adjusted *p-value* < 0.05) between bM2-like TAMs and mM2-like TAMs were ordered by *|*log*2FC|*, and GSEA was performed using the gseKEGG function of R package clusterProfiler version 4.2.2 (Wu et al., 2021) to further understand the difference in biofunction between the two M2-like TAMs subclusters, confirming the results of KEGG analysis.

### 2.7 Cell-cell communication analysis with CellChat

CellChat analysis was performed based on all immune cells of the primary group to depict the cell-cell crosstalk profiles of immune cells in PDAC TME. Cell-cell communication analysis was performed using the R package CellChat version 1.1.3 (Jin et al., 2021) to infer the possible mechanisms of the prognosis-improving phenotype of patients with high amount of bM2-like TAMs. The normalized matrix of the Seurat object was converted into a CellChat object, and a biologically significant cell-cell communication network in which the *p*-value of a single pathway was less than 0.05 was constructed based on the expression of known ligand-receptor pairs. We adjusted the *p-value* based on the pipeline previously reported (Jin et al., 2021). Furthermore, the difference in communication strength with other cell types between bM2-like TAMs and mM2-like TAMs was compared. Since cell receptor analysis was affected by the large differences in tumor microenvironment between primary and metastatic tumors, we only analyzed primary tumor samples here.

### 2.8 Analysis of bulk RNA-Seq data

Kaplan–Meier survival analysis was generated by implementing the R package survival version 3.2–13 pipeline. Briefly, the cutoff values were selected using the X-tile software. The 172 patients from the TCGA dataset were divided into two groups based on their expression of SLC12A5 and ENPP2 (patients with top 70% expression of both genes were divided into “high” group, and the rest of the patients were in “low” group; n (high) = 96, n (low) = 76), and the survival analysis was performed to identify the impact of the expression of the two genes on prognosis.

To explore the impact of bM2-like TAMs on the PAAD stage, 172 PAAD patients were divided into three groups to reduce the contingencies associated with simply splitting into two groups. Patients whose expression of SLC12A5 and ENPP2 were both in the top 50% were divided into bM2-like-TAMs-high group (bTAMs_high), both in the bottom 50% were identified as bM2-like-TAMs-low group (bTAMs_low), and the remaining patients were identified as bM2-like-TAMs-medium group (bTAMs_medium) (n (bTAMs_high) = 60, n (bTAMs_medium) = 58, n (bTAMs_low) = 60). The R package GSVA version 1.50.0 was used to perform the ssGSEA analysis on the RNA-seq expression matrix with KEGG gene sets as reference.

### 2.9 Analysis of gene chip data

Survival analysis of gene chip data from the pancreatic cancer cohort of more than one thousand patients was performed using Kalan Meier Plotter (https://kmplot.com/analysis/) (Posta and Győrffy, 2023) The gene chip data were obtained from GEO, the parameter was set to its default value (Posta and Győrffy, 2023). The cutoff value was automatically selected where the *p-value* is most significant.

### 2.10 Immunohistochemistry (IHC)

IHC data were obtained from the Human Protein Atlas (HPA) database (https://www.proteinatlas.org/). The expression of ENPP2 (HPA023700) was compared between normal pancreatic tissue and pancreatic cancer tissue. The correlations of protein expression levels of CHTI1 (HPA074844), ENPP2 (HPA023700) and ACTA2 (HPA041271) were analyzed between pancreatic cancer patients.

### 2.11 Code availability

All related code is available on GitHub. https://github.com/Wangzzzzzzzzz/PDAC_code.

## 3 Results

### 3.1 Single-cell transcriptomics revealed the heterogeneity of PDAC tumor microenvironment

**FIGURE 1:**
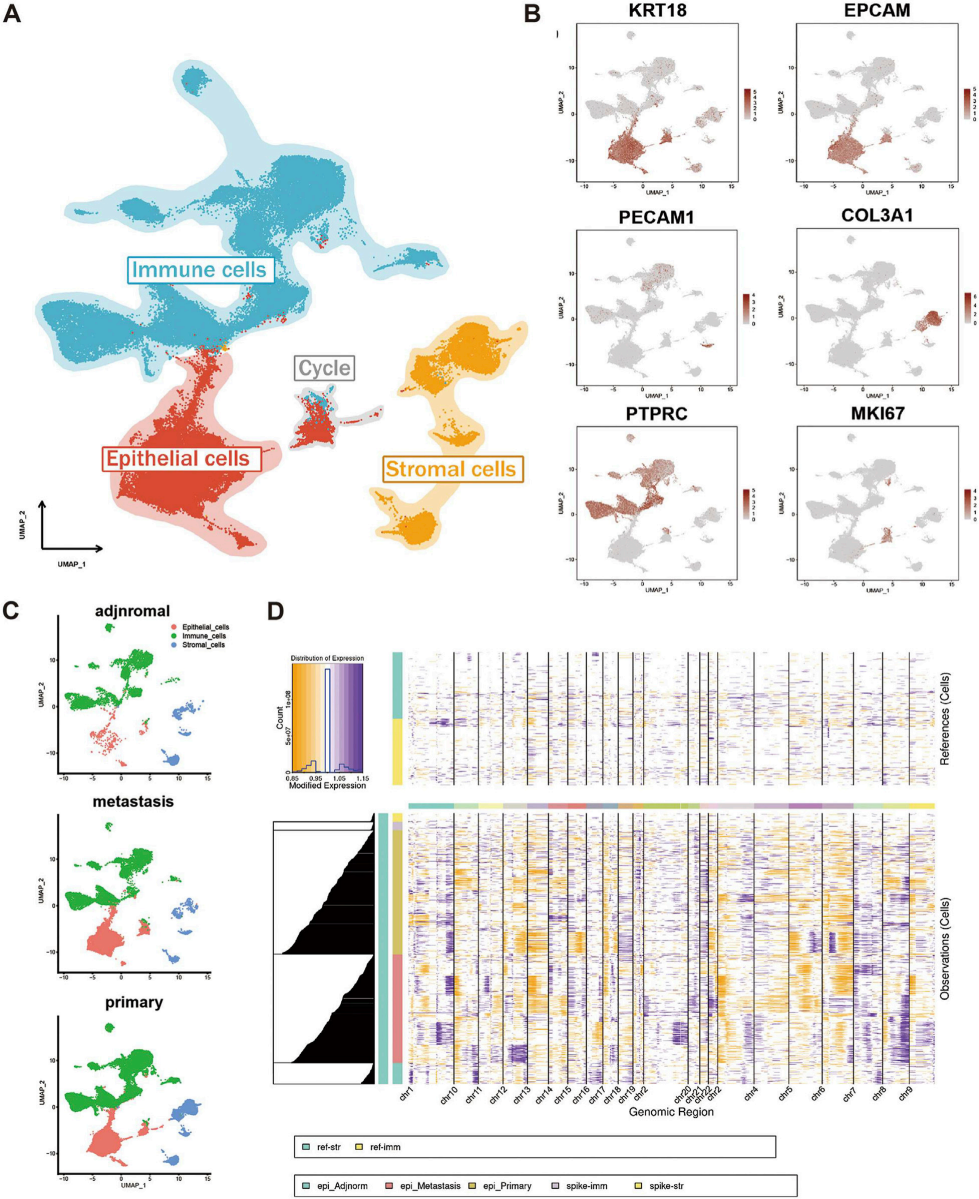
Preprocessing and inferCNV analysis of PDAC scRNA-seq data. **(A)** UMAP visualization of scRNA-seq data which were generally divided into 4 cell types. Blue is immune cells, red is epithelial cells, yellow is stromal cells and gray is the cells that are in the cell cycle. **(B)** Markers for epithelial cells (KRT18 and EPCAM), stromal cells (PECAM1 and COL3A1), immune cells (PTPRC) and cells that are in the cell cycle (MKI67). **(C)** Cell quantity of adjacent samples (top), metastatic samples (middle) and primary tumor samples (bottom). Displayed in UMAP. **(D)** Visualization of the inferCNV analysis. In contrast to immune cells and stromal cells, purple areas indicate increased chromosome copy number and yellow areas indicate decreased chromosome copy number in epithelial cells.

The scRNA-seq data of 40 samples were obtained from the GEO database, consisting of 27 primary tumor samples, 10 metastasis samples, and 3 adjacent normal samples (Table 1). After preprocessing, a total of 80,273 cells were grouped into 37 clusters, which were generally divided into 4 cell types for further analysis (Figure 1A; Supplementary Figure S1A). The cell types were defined by typical cell markers as indicated in previous studies (Tirosh et al., 2016; Lin et al., 2020): epithelial cells (KRT18 and EPCAM), stomal cells (PECAM1 and COL3A1), immune cells (PTPRC), and cells undergoing the cell cycle (MKI67) (Figures 1B, C). The fractions of three cell types were found to be variable in different groups (Supplementary Figure S1B). By calculating large-scale chromosomal CNV for each cell type using inferCNV, we found that epithelial cells had significantly higher CNV levels than immune cells and stromal cells (Figure 1D). There were also a few CNV events in adjacent normal epithelial cells, which may be related to acinar ductal metaplasia (ADM) or a small lesion in the adjacent tissue. Acinar cells and normal ductal cells are in the process of becoming malignant tumor cells (Ferreira et al., 2017). In general, inferCNV analysis showed that epithelial cells had different degrees of malignancy, and it was also reported that epithelial cells were divided into high malignant cells and low malignant cells for analysis (Fang et al., 2022). In fact, the distributions of epithelial cells are different in different samples, which have different pathway activities. It has been reported that the number of receptor-ligand interactions between immune cells and epithelial cells increases during the evolution from normal cells to malignant cells (Jiang et al., 2021), suggesting that malignant cells might educate immune cells into tumor-promoting cells through cell-cell communication. Therefore, the changes in the immune microenvironment in heterogeneous tumors and the crosstalk between immune cells and malignant cells deserve further analysis.

### 3.2 bM2-like TAMs defined by SLC12A5 and ENPP2 was associated with PAAD prognosis

**FIGURE 2:**
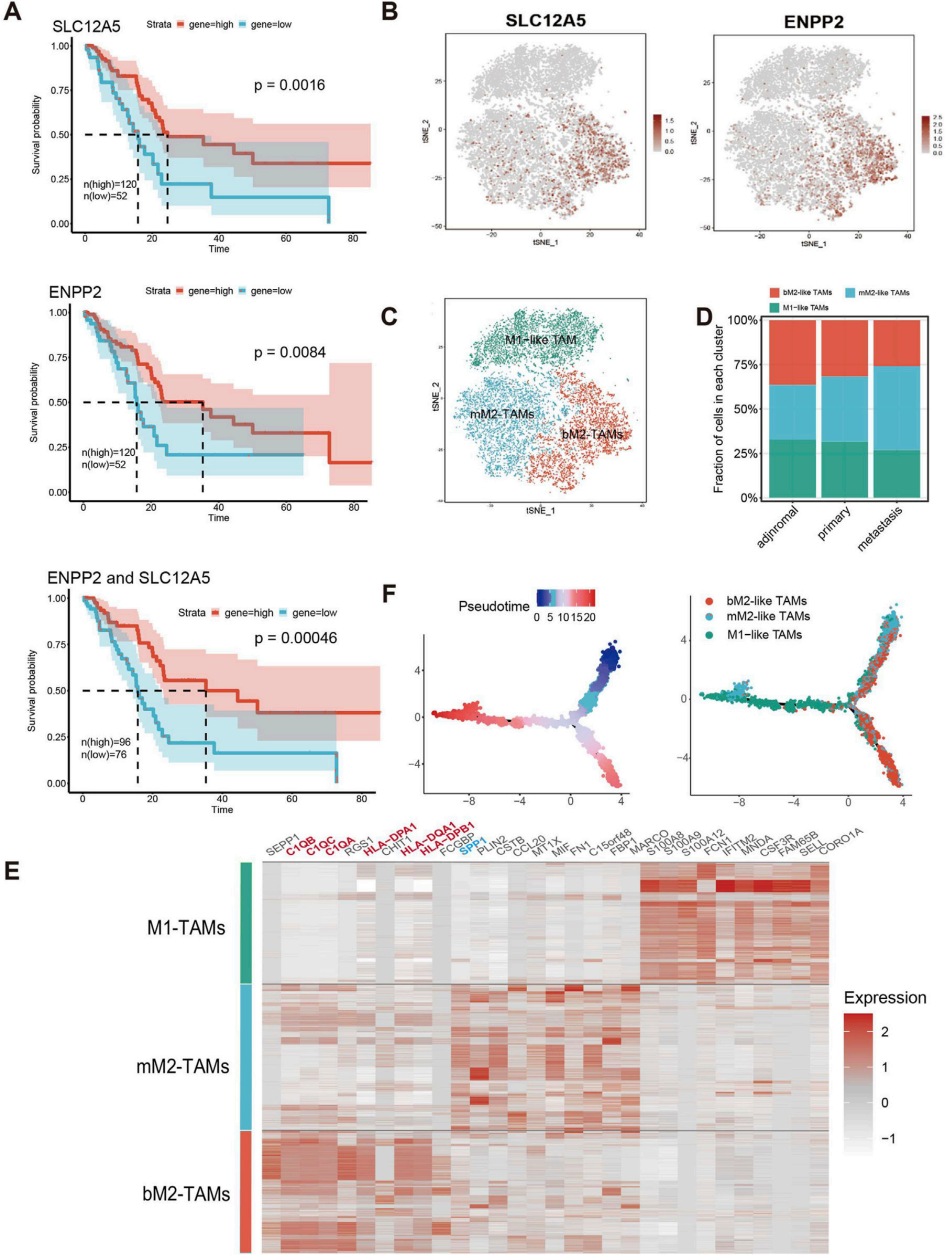
bM2-like TAMs were a TAM subclusters defined by co-expression of SLC12A5 and ENPP2. **(A)** Survival analysis of SLC12A5 (top), ENPP2 (middle) and co-expression of both genes (bottom) in the TCGA PAAD cohort. (n (high) = 120, n (low) = 52, *p* (SLC12A5) = 0.0016, *p* (ENPP2) = 0.0084, *p* (ENPP2 and SLC12A5) = 0.00046). **(B)** Expression of SLC12A5 and ENPP2 in TAMs in scRNA-seq data, visualized by t-SNE. **(C)** t-SNE projection of TAMs. TAMs were subclustered into bM2-like TAMs, mM2-like TAMs, and M1-like TAMs according to their inferred cell type identities. **(D)** Fraction of TAMs subclusters in adjacent normal, tumor metastasis and primary tumor groups. **(E)** Heatmap of DEGs in three TAMs subclusters. **(F)** Pseudotime trajectory analysis of TAMs.

Using bulk RNA-seq data of 172 PAAD patients from the TCGA database, we identified two correlated genes whose expression profiles were closely associated with PAAD prognosis (Figure 2A; Supplementary Figure S1C). Specifically, based on t-SNE reduction results on scRNA-seq data, TAMs were revealed to consist of three subclusters (M1, M2-1 and M2-2). For better definition of two M2 TAMs subclusters, we found two prognosis-associated genes based on the following process: DEGs between the M2-1 and M2-2 subclusters were identified in the scRNA-seq data, and the genes with *p-value* less than 0.05 and *|*log*2FC|* more than 0.2 were reserved for further analysis (572 genes in total). Univariate Cox hazard analysis was performed on 572 DEGs using bulk RNA-seq data from the TCGA database of 172 PAAD patients, and 62 genes were found to be prognostically favorable (*p* < 0.1 and *HR* < 1). Among the 62 genes, only two genes, SLC12A5 and ENPP2, were mainly expressed in the M2-1 subcluster (Supplementary Figure S1D). SLC12A5 (also known as KCC2), an integral membrane potassium-chloride cotransporter mainly involved in maintaining chloride homeostasis in neurons, has recently been reported as a potential prognostic biomarker for human cancer (Jiang et al., 2021). ENPP2, also known as ATX, encodes a secreted enzyme that functions as both a phosphodiesterase and a phospholipase. Remarkably, high expression of both genes was significantly associated with prolonged patient survival (SLC12A5, *p-value* = 0.0016; ENPP2, *p-value* = 0.0084) (Figure 2A). The significance of this effect was further enhanced in patients with concurrent high expression of both SLC12A5 and ENPP2 (Figure 2A).

As expected, at the scRNA-seq level, the co-expression of the two prognosis-related genes was mainly observed in a specific group of immune cells (Supplementary Figure S1E). The immune cells were extracted, grouped by t-SNE, and then annotated (Supplementary Figure S1F). Notably, SLC12A5 and ENPP2 were shown to be co-expressed in a subcluster of M2-like TAMs (Figures 2B, C; Supplementary Figure S1G), which were defined as benign M2-like TAMs (bM2-like TAMs). Conversely, M2-like TAMs with low expression of the two genes were defined as malignant M2-like TAMs (mM2-like TAMs). The observed significant improvement in prognosis of PAAD patients with high expression levels of the two genes suggested a potential positive influence of bM2-like TAMs on prognosis. To investigate this hypothesis, we compared cell fractions from three different TAMs subclusters in the scRNA-seq data. As expected, the number of mM2-like TAMs increased with advancing tumor stage, while the number of bM2-like TAMs showed the opposite trend, further indicating the positive prognostic impact of bM2-like TAMs in PDAC TME (Figure 2D).

The genes upregulated in bM2-like TAMs, including C1QB, C1QC, C1QA, HLA-DPA1, HLA-DQA1, and HLA-DPB1, were found to be related to the complement pathway and antigen presentation (Figure 2E). C1QC+ TAMs were reported to play a tumor suppressive role in colorectal cancer and its liver metastasis through phagocytosis and antigen presentation (Zhang et al., 2020; Liu et al., 2022b). Meanwhile, SPP1+ TAMs have been proved to be characterized by their tumor promoting activities and are associated with metastasis in colorectal cancer (Zhang et al., 2020). Similar phenomena have also been reported in PDAC (Werba et al., 2023). Notably, consistent with previous study, C1QC was among the top 10 highly-expressed genes in bM2-like TAMs, while SPP1 was upregulated in mM2-like TAMs (Figure 2E; Supplementary Figure S1H). Other genes confirmed to be expressed in TAMs with anti-tumor and favorably prognostic functions, CXCL9 (Bill et al., 2023) and CD169, were also enriched in bTAMs (Supplementary Figure S1H), further indicating the tumor suppressive properties of bM2-like TAMs.

As widely reported, M1-like TAMs and M2-like TAMs exhibit high plasticity and can be converted into each other under certain conditions (Pan et al., 2020). M1-like TAMs mainly act as tumor suppressors through proinflammatory effects mediated by multiple mechanisms involving the release of ROS and NO. In contrast, M2-like TAMs facilitate tumor progression by promoting metastasis, angiogenesis, and tumor cell proliferation (Pan et al., 2020). Thus, we hypothesized that bM2-like TAMs represent a group of macrophages with a lower degree of polarization, exhibiting an intermediate level of differentiation between M1-like TAMs and M2-like TAMs. To our surprise, the results of pseudotime trajectory analysis suggested that the bM2-like TAMs were a group of highly polarized M2-like TAMs (Figure 2F).

Overall, we used two favorable prognosis-related genes to define a highly differentiated subcluster as bM2-like TAMs that may improve prognosis by regulating the TME of PDAC.

### 3.3 bM2-like TAMs improve PDAC prognosis by regulating TME

**FIGURE 3:**
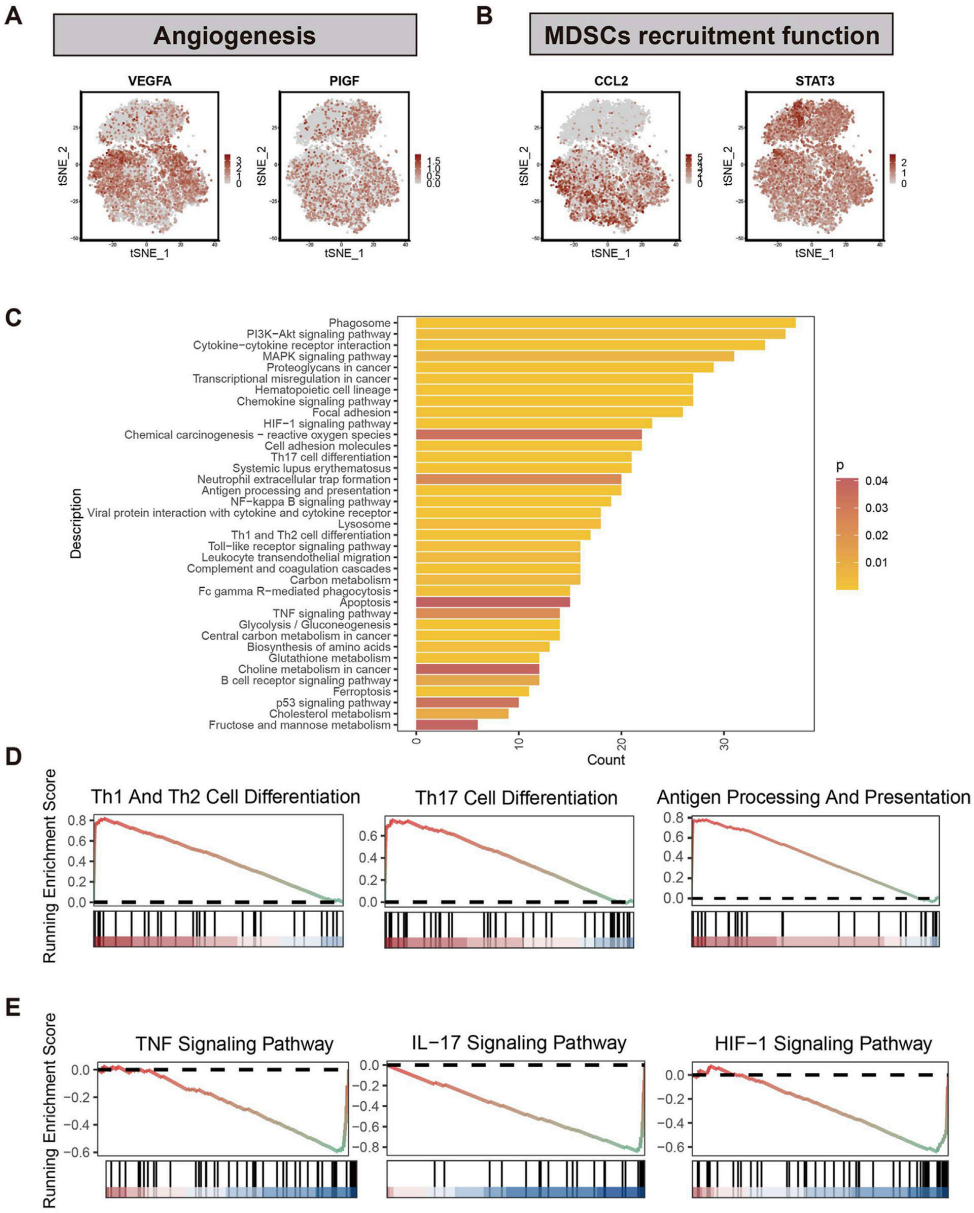
Differential analysis of two subclusters of M2-like TAMs. **(A)** Expression profiles of genes associated with angiogenesis (VEGFA and PIGF) and **(B)** myeloid-derived suppressor cells (CCL2 and STAT3). **(C)** Results of KEGG enrichment analysis based on the DEGs between bM2-like TAMs and mM2-like TAMs. Different colors represent different *p-values*, from red to yellow indicates that *p-values* from large to small, and the enrichment results are more and more significant. **(D)** GSEA analysis using the KEGG dataset revealed the pathways that were upregulated in bM2-like TAMs and **(E)** mM2-like TAMs.

Given that M2-like TAMs are primarily associated with a tumor-promoting role in TME, we examined the distribution of genes associated with angiogenesis (VEGFA and PIGF (Jetten et al., 2014)) and myeloid-derived suppressor cells (MDSCs) recruitment to compare the tumor-promoting functions of three TAMs subclusters in the scRNA-seq data (Figures 2C, 3A, B). Notably, the expression of genes in bM2-like TAMs was not enriched, indicating that tumor-promoting functions of bM2-like TAMs were not enhanced compared to other TAMs subclusters.

To further explore the prognosis-associated properties of bM2-like TAMs, differential analysis was performed in the scRNA-seq data to find out the differences between bM2-like TAMs and mM2-like TAMs. The DEGs between the two subclusters of M2-like TAMs were calculated, and their expression profiles were subjected to KEGG enrichment analysis (Figure 3C). As shown by the enrichment results, the two subclusters showed distinct expression patterns in tumor-associated pathways, including PI3K-Akt pathway, MAPK pathway, chemokine pathway, HIF-1 pathway, NF-κB pathway, and TNF pathway. In addition, bM2-like TAMs and mM2-like TAMs also differ in pathways associated with helper T cell differentiation, suggesting the diverse roles of the subclusters in TME regulation. GSEA was performed to further investigate the discrepancy of two subclusters of M2-like TAMs. Compared with mM2-like TAMs, pathways associated with helper T cell activation, antigen-presenting and cell adhesion molecules were upregulated in bM2-like TAMs (Figure 3D; Supplementary Figure S2A), whereas TNF, IL-17 and HIF-1 pathways showed an opposite tend (Figure 3E). Thus, we speculated that bM2-like TAMs might regulate PDAC TME by activating T cells and consequently lead to improved prognosis.

### 3.4 bM2-like TAMs activate T cells through ALCAM/CD6

**FIGURE 4:**
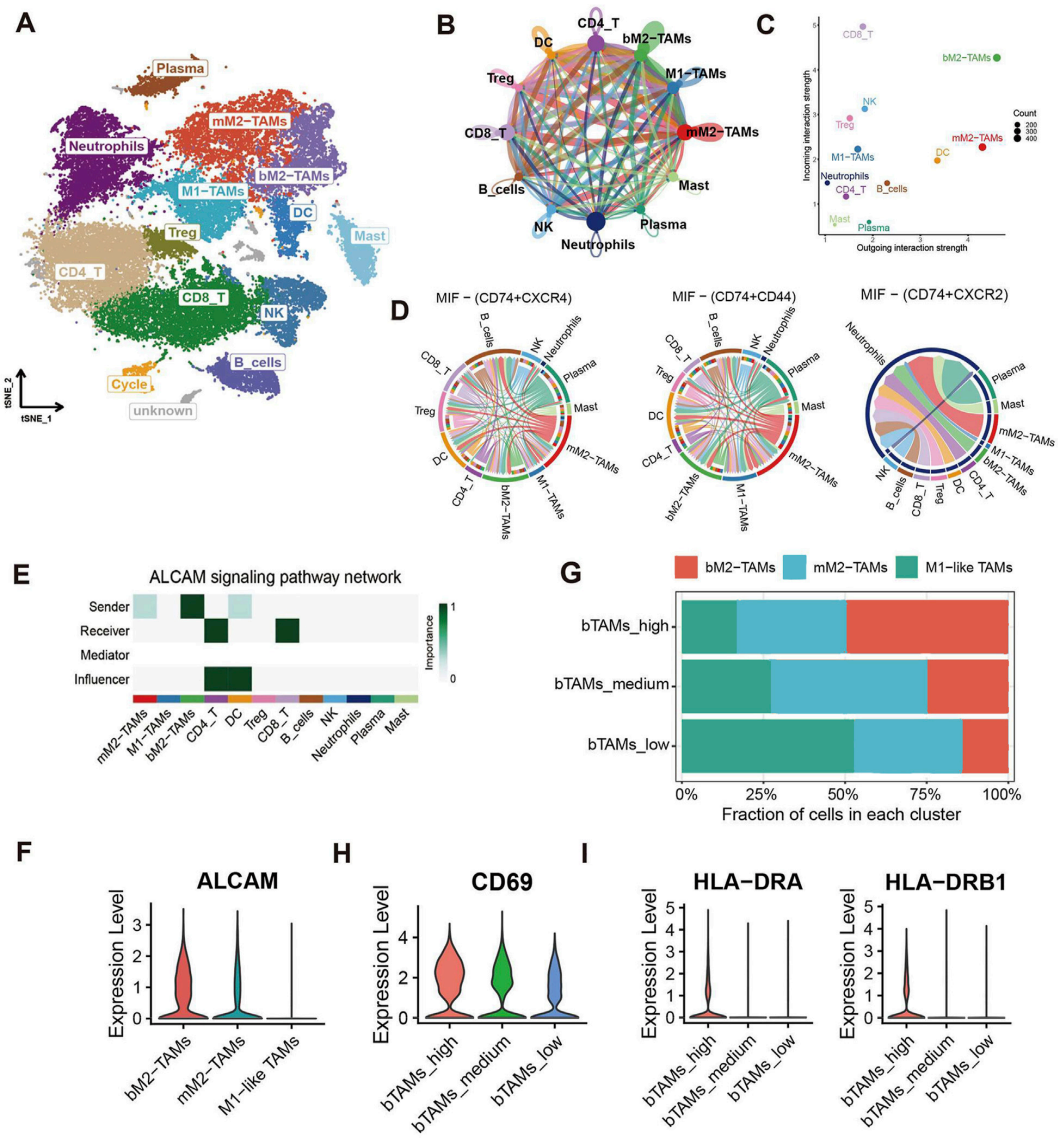
CellChat analysis of M2-like TAMs and other immune cells. **(A)** t-SNE visualization of immune cell subclusters. **(B)** Number of interactions identified by the CellChat R package. The width of the arrows indicates the number of interactions, and the arrow of the same color represents the output signals of a single immune cell subcluster. **(C)** Incoming and outgoing interaction strength of immune cell subclusters. **(D)** Chord diagram of the MIF pathway. **(E)** Pathway network visualization of the ALCAM signaling pathway. **(F)** Violin plot of ALCAM in 3 TAMs subclusters. **(G)** Cell fractions of TAMs subclusters in bTAMs_high, bTAMs_medium and bTAMs_low groups. Violin plot showed the expression of **(H)** CD69 and **(I)** HLA-DR in patients with high, medium or low amount of bM2-like TAMs.

To further investigate how bM2-like TAMs regulate PDAC TME, CellChat analysis was performed based on all immune cells of the primary group to depict the cell-cell crosstalk profiles of immune cells in PDAC TME (Figures 4A, B). Compared with M1-like TAMs and mM2-like TAMs, bM2-like TAMs showed higher interaction strength (Figure 4C), which may indicate a more active immune regulatory property of bM2-like TAMs in PDAC TME.

Macrophage migration inhibitory factor (MIF), which is mainly produced by both malignant cells and infiltrating leukocytes, has been reported to play a tumor-promoting role in human cancers (Candido and Hagemann, 2013). The results of CellChat analysis revealed an increased output signal of the MIF pathway in mM2-like TAMs, which may contribute to the tumor-promoting property of mM2-like TAMs, while bM2-like TAMs expressed MIF at a relatively low level (Figure 4D). In addition, upregulation of the SPP1 signaling pathway was also observed in mM2-like TAMs (Supplementary Figure S2B), with the poor prognosis-associated characteristics of SPP1 in most cancers including PAAD, as previously reported (Liu et al., 2022c).

On the contrary, pathways with favorable influence on prognosis, including CADM pathway, complement pathway, galectin pathway and SN pathway, were mainly enriched in bM2-like TAMs (Supplementary Figure S2C–F). Notably, the output signal of the activated leukocyte cell adhesion molecule (ALCAM) pathway was significantly increased in bM2-like TAMs compared with mM2-like TAMs (Figures 4E, F). ALCAM, also known as CD166, mainly expressed on antigen-presenting cells, is the ligand of CD6, the co-stimulatory receptor expressed on T cells. The ALCAM-CD6 interaction has been demonstrated to be associated with T cell activation and proliferation (Zimmerman et al., 2006; Hassan et al., 2004).

**FIGURE 5:**
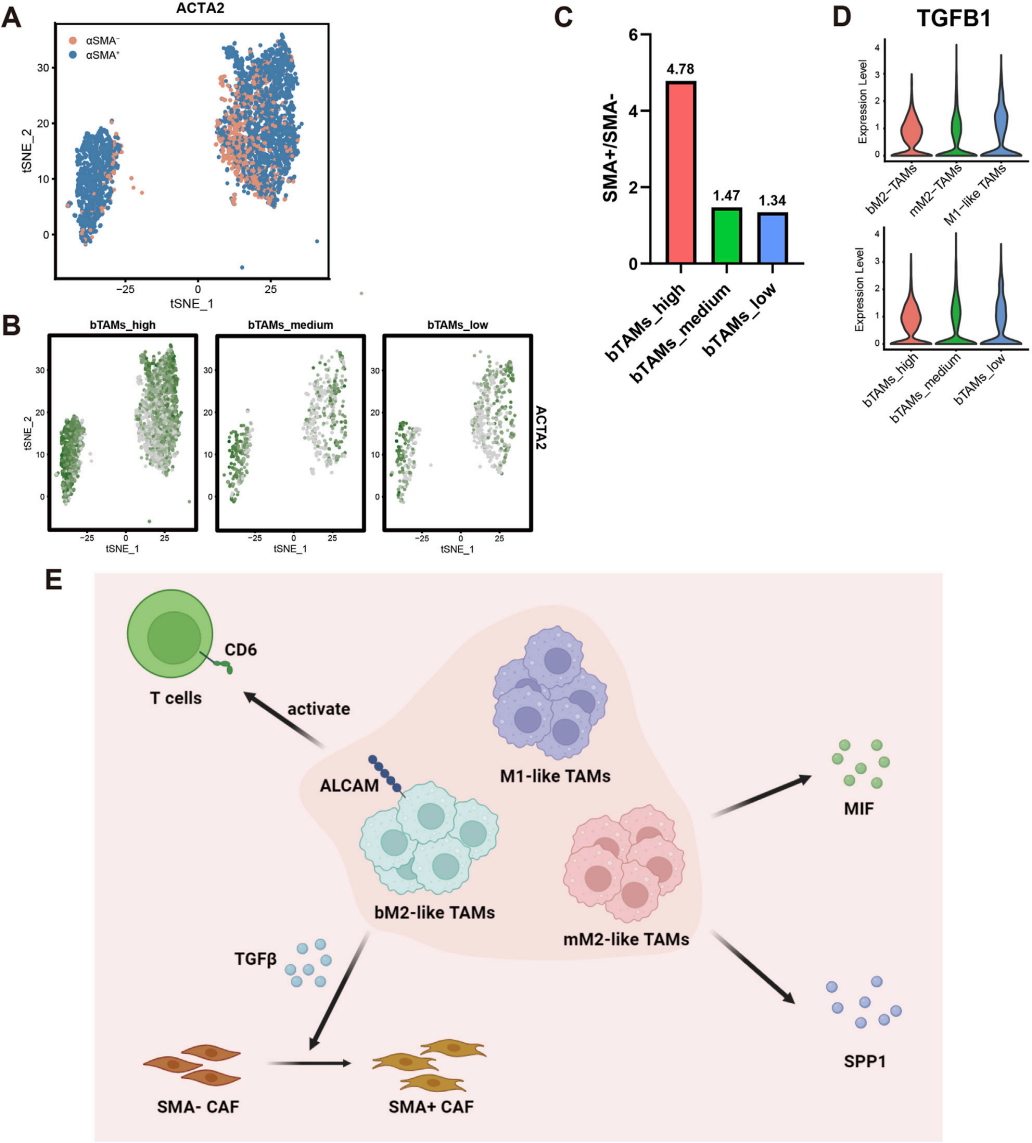
Number of α-SMA+ myofibroblasts in patients with different fractions of bM2-like TAMs. **(A)** Cell clusters of stromal cells were visualized by t-SNE. PSCs and CAFs were annotated as α-SMA+ cells or α-SMA-cells according to their expression of ACTA2. **(B)** Number of PSCs and CAFs in patients with high, medium, or low bM2-like TAMs fraction. The shading corresponds to expression intensity of ACTA2. **(C)** Ratio of α-SMA+ cells to α-SMA-cells in bTAMs_high, bTAMs_medium and bTAMs_low groups. **(D)** Expression of TGFβ in 3 TAMs subclusters (top) and in patients with high, medium or low bM2-like TAMs fraction (bottom). **(E)** bTAMs associated with better prognosis through T cell activation and stroma remodeling.

To further investigate whether bM2-like TAMs could enhance T cell activation through increased ALCAM-CD6 interaction, the scRNA-seq samples were divided into 3 groups (n (bTAMs_high) = 13, n (bTAMs_medium) = 13, n (bTAMs_low) = 14) according to the fraction of bM2-like TAMs within total TAMs (Figure 4G; Table 1). The proportion of mM2-like TAMs showed little change within three groups, possibly indicating the relatively independent differentiation pattern of mM2-like TAMs. Next, we observed the expression of T cell activation markers in the three groups. As expected, CD69, the early-stage activation marker of T cells, was found to have the highest expression level in the bTAMs_high group and the lowest expression level in the bTAMs_low group (Figure 4H). And the marker of late activation of T cells, HLA-DR, were found to be expressed only in bTAMs_high group (Figure 4I), confirming the hypothesis that bM2-like TAMs were likely to enhance the activation of T cells. Therefore, while mM2-like TAMs create an immunosuppressive phenotype of PDAC through the MIF and SPP1 pathways, bM2-like TAMs might promote T cell activation through the ALCAM-CD6 interaction (Figure 5E).

### 3.5 bM2-like TAMs generate prognosis-favorable αSMA+ myofibroblasts through secreting TGFβ

Surrounded by an abundant and dense collagenous stroma, PDAC is thought to have a fibrotic TME that is largely formed by stromal cells. PDAC stromal cells are predominantly composed of pancreatic stellate cells (PSCs), cancer-associated fibroblasts (CAFs) and a progenitor population of CAFs. Knowing that deletion of α-SMA+ myofibroblasts in mice leads to increased tumor invasion and worse prognosis (Özdemir et al., 2014), we investigated the proportion of α-SMA+ myofibroblasts in the bTAMs_high, bTAMs_medium and bTAMs_low groups. All stromal cells were extracted and annotated, and based on the annotation results, PSCs and CAFs were extracted and divided into α-SMA+ cells and α-SMA- cells according to the expression of ACTA2, the gene encoding α-SMA (Figure 5A). Although the total number of PSCs and CAFs was significantly increased in the bTAMs_high group (Figure 5B), the ratio of α-SMA+ cells to α-SMA- cells was higher in the bTAMs_high group (4.78) than in the bTAMs_medium (1.47) and bTAMs_low (1.34) groups (Figure 5C), suggesting that bM2-like TAMs may evolve in PDAC stroma formation by promoting the differentiation of α-SMA+ cells while suppressing the formation of α-SMA- cells. TGFβ has been reported to be responsible for the terminal differentiation of α-SMA+ cells (Evans et al., 2003; Vaughan et al., 2000; Biffi et al., 2019). We analyzed the expression of TGFβ in TAMs and the expression of downstream effectors in PSCs and CAFs (Figure 5D; Supplementary Figure S2G–H). TGFβ was upregulated in both bM2-like TAMs and bTAMs_high group (Figure 5D). Upregulation of TGFβ effector, including SMAD3, SMAD5 and CTGF, was observed in PSCs and CAFs in bTAMs_high group (Supplementary Figure S2H). Taken together, our analysis revealed that bM2-like TAMs could alter the stromal cell composition by promoting the differentiation of α-SMA+ myofibroblasts through the TGFβ signaling pathway (Figure 5E).

### 3.6 The prognosis-favorable characteristic of bM2-like TAMs and its influence on T cells and stromal cells

**FIGURE 6:**
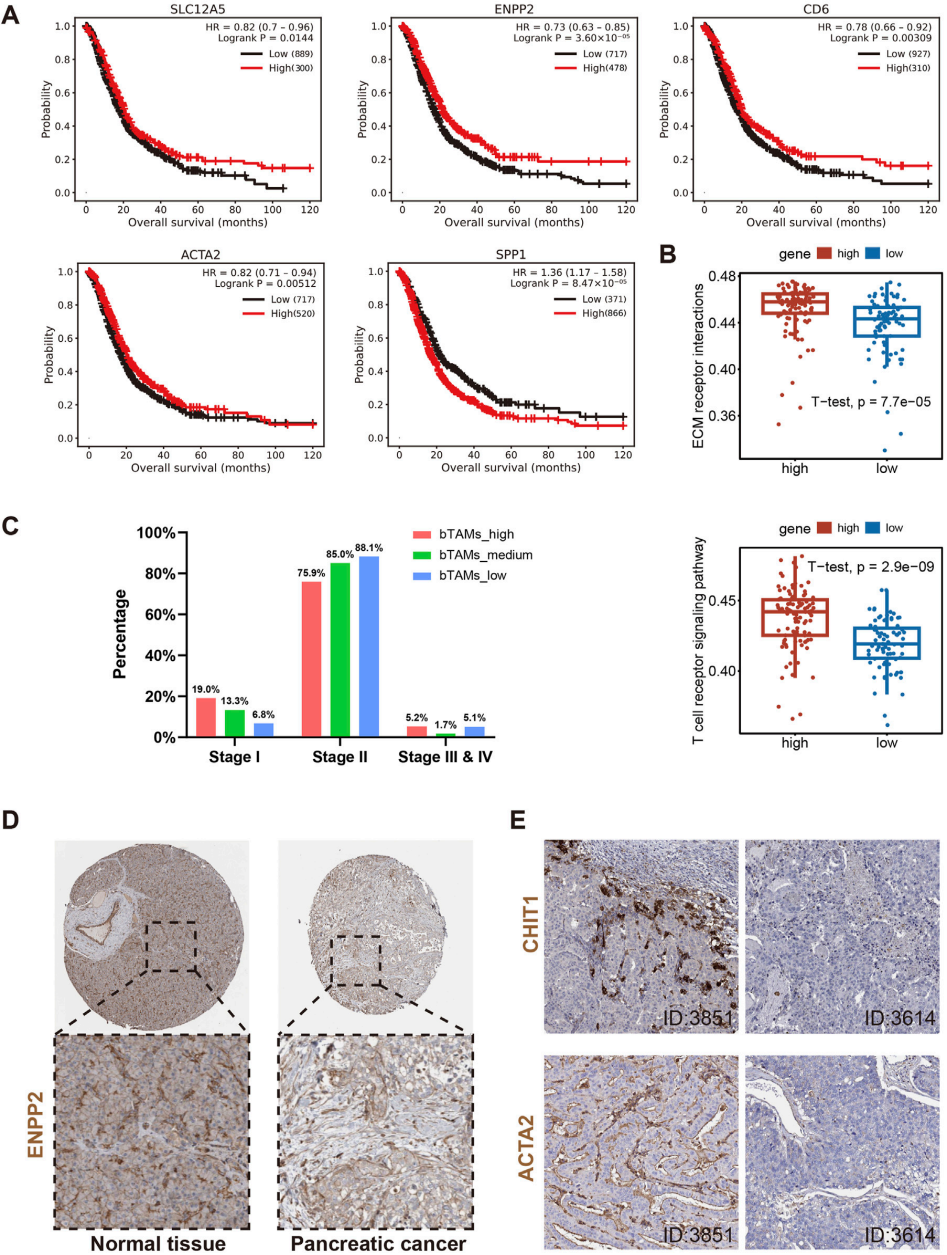
Further validation of the above hypotheses using gene chip, bulk RNA-seq and IHC data. **(A)** Survival analysis of genes associated with definition of bM2-like TAMs (SLC12A5 and ENPP2), activation of T cells (CD6) and definition of α-SMA+ cells (ACTA2), and gene upregulated in mM2-like TAMs (SPP1). **(B)** ssGSEA analysis of bulk RNA-seq from TCGA dataset revealed upregulation of ECM receptor interaction and T cell receptor signaling pathway in patients with higher co-expression of SLC12A5 and ENPP2, *p-value* = 7.7*10^5 and 2.9*10^9, respectively. **(C)** Classification of the PAAD patients into three groups based on the expression of SLC12A5 and ENPP2 (n (bTAMs_high) = 60, n (bTAMs_medium) = 58, n (bTAMs_low) = 60). The number of bM2-like TAMs in different stages of PAAD was counted. **(D)** Expression of protein encoded by ENPP2 in normal pancreatic tissue and pancreatic cancer tissue. **(E)** Protein expression of CHIT1 and ACTA2 in PAAD tissues, patient IDs in HPA were 3851 (left) and 3614 (right).

Gene chip datasets of pancreatic cancer were obtained from the Kalan Meier Plotter database (Posta and Győrffy, 2023) (https://kmplot.com/analysis/). Survival analysis of gene chip data confirmed the prognostic property of genes associated with bM2-like TAMs and their immunoregulatory activities (Figure 6A), while genes involved in the biofunction of mM2-like TAMs were found to be negatively correlated with overall survival (Figure 6B).

The ssGSEA analysis was performed on RNA-seq data of 172 PAAD patients from the TCGA database. The ssGSEA result showed that patients with more bM2-like TAMs had higher enrichment scores in pathways related to antigen processing and presentation, leukocyte transendothelial migration, T cell receptor signaling, and ECM receptor (Figure 6B; Supplementary Figure S3A), which was consistent with previous literature (Zhang et al., 2020) and our analyses. To investigate the association between bM2-like TAMs and PAAD stage, we divided PAAD patients into three groups based on the expression of SLC12A5 and ENPP2 (n (bTAMs_high) = 60, n (bTAMs_medium) = 58, n (bTAMs_low) = 60). The percentage of stage I patients was positively correlated with the proportion of bM2-like TAMs, while a sequential increase in the percentage of stage II patients was observed in the three groups, which might indicate the indicative role of bM2-like TAMs in tumor staging (Figure 6C). Furthermore, the expression of genes involved in T cell activation and myofibroblast differentiation, CD69, HLA-DRA, HLA-DRB1, and ACTA2, also showed a decreasing trend with the decrease of the bM2-like TAMs fraction (Supplementary Figure S3B).

IHC data were obtained from the HPA dataset available at proteinatlas.org. Compared to normal pancreatic tissue, pancreatic tumor tissue showed lower ENPP2 expression (Figure 6D). Knowing that the protein encoded by CHIT1 was mainly expressed in bM2-like TAMs (Supplementary Figure S3C), to some extent, the expression level of CHIT1 is positively associated with the amount of bM2-like TAMs. The IHC data revealed that in patients with higher expression of CHIT1, the expression level of ACTA2 was also significantly increased (Figure 6E), consistent with our suggestion that the number of α-SMA+ myofibroblasts and bM2-like TAMs have a positive correlation. In addition, the expression of ENPP2 and ACTA2 was also positively correlated (Supplementary Figure S3D).

## 4 Discussion

Due to the high heterogeneity and complexity of the pancreatic tumor microenvironment, immunotherapy has not emerged as a major therapeutic option for PDAC and has failed to improve overall survival (Somani et al., 2022; Raghavan et al., 2021; Guo et al., 2021). Therefore, a thorough understanding of the composition and function of the TME is critical to the development of potential immunotherapeutic strategies for PDAC. In this study, by integrating and analyzing scRNA-seq and bulk RNA-seq data, we defined M2-like TAMs with high expression of the prognostically favorable genes SLC12A5 and ENPP2 as bM2-like TAMs with antitumor properties. The identification of this novel subtype can be attributed, at least in part, to the availability of sufficient datasets. As a highly differentiated M2-like TAMs subset, bM2-like TAMs may play an anti-tumor role by activating T cells via ALCAM/CD6 interactions, as well as by promoting α-SMA+ myofibroblasts production via TGF-β secretion. Our findings emphasize the phenotypic and functional heterogeneity of M2-like TAMs in PDAC.

Due to its complexity, the exact mechanism of how PDAC TME regulates the immune response is not well understood. TAMs serve as a critical component in PDAC TME, and are reported to be responsible for promoting tumor progression (Pan et al., 2020). Recently, the high-resolution scRNA sequencing technique has been widely used to explore the biological function and intertumoral heterogeneity of TAMs, and several TAMs subclusters have been identified (Polani et al., 2021). C1QC+ TAMs, characterized by the upregulation of pathways involved in phagocytosis and antigen presentation, are thought to exhibit pro-inflammatory and anti-tumor activities in colorectal cancer (CRC) and CRC liver metastasis (CRLM) (Zhang et al., 2020). In another study, CXCL9+ TAMs were described as TAMs associated with favorable prognosis in several cancers (Bill et al., 2023). In contrast, SPP1+ TAMs have been described to be associated with angiogenesis and metastasis and negatively correlated with overall survival (Zhang et al., 2020; Bill et al., 2023). C1QC+ TAMs and SPP1+ TAMs have also been identified in PDAC, and were found to share similar characteristics with those in other human cancers (Bill et al., 2023). In this study, our newly defined bM2-like TAMs with high expression of SLC12A5 and ENPP2 share certain similarities with C1QC+ TAMs and CXCL9+ TAMs, while the remaining mM2-like TAMs share certain similarities with SPP1+ TAMs. *Bill R* et al. found that macrophage polarity, defined by CXCL9 and SPP1 (CS) expression, had a noticeably strong prognostic association in head and neck squamous cell carcinomas (Bill et al., 2023). CS TAM polarity identifies TME-wide coordination of pro- and anti-tumor pathways. In this study, bM2-like TAMs defined by SLC12A5 and ENPP2 was associated with PAAD prognosis. Overall, these TAMs are not defined by the conventional M1 and M2 markers and have different modalities of regulation, reflecting the complexity of the human tumor microenvironment.

ALCAM and CD6 are cell surface proteins involved in regulating immune responses (te Riet et al., 2014). ALCAM-CD6 interaction can promote T cell-mediated anti-tumor immunity by enhancing the recruitment and activation of effector T cells (Cho et al., 2023). Additionally, ALCAM-CD6 signaling can modulate the balance between pro-inflammatory and regulatory T cells, potentially influencing the overall immune response to tumors (Kenney et al., 2023). Based on scRNA-seq, we demonstrated a crosstalk between bM2-like TAMs and T cells via the ALCAM-CD6 interaction, which may at least partially explain the anti-tumor role of this newly identified TAMs subset. Consistently, several genes involved in T cell activation, such as CD69, were also found to be positively associated with the infiltration of bM2-like TAMs in PDAC. Differently, mM2-like TAMs may promote PDAC progression by secretion of MIF and SPP1.

Both primary pancreatic tumors and metastases are characterized by a desmoplastic stroma that accounts for approximately 80% of tumor volume (Guo et al., 2023). We found that both bM2-like TAMs and mM2-like TAMs were present in both primary tumor and metastatic tumor samples. In the metastatic tumor samples, the number of mM2-like TAMs increased with advancing tumor stage, while the number of bM2-like TAMs showed the opposite trend, further indicating the positive prognostic impact of bM2-like TAMs in PDAC TME. The mechanisms underlying the formation of fibrotic stroma in PDAC are not fully understood. TGF-β axis is recognized as one of the most commonly altered signal transduction pathways in pancreatic cancer, and play a key role in fibrotic stroma formation (Zhan et al., 2015; Hussain et al., 2018). Based on scRNA-seq, we currently demonstrated that bM2-like TAMs may be involved in PDAC fibrotic stroma remodeling by regulating the α-SMA+/α-SMA-ratio in PSCs and CAFs through secretion of TGF-β. Survival analysis of gene chip data further indicated the positive impact of bM2-like TAMs on overall survival. ssGSEA analysis of bulk RNA-seq revealed the enrichment of ECM receptor, antigen processing and presentation, T cell receptor signaling, and leukocyte transendothelial migration pathway in patients with higher levels of bM2-like TAMs. In addition, the abundance of bM2-like TAMs in patients was also found to be associated with PAAD stage. ENPP2 was less expressed in pancreatic cancer tumor tissues compared to normal tissues, suggesting that the decrease of bM2-like TAMs may be pathological. The positive correlation of bM2-like TAMs and α-SMA+ myofibroblasts was confirmed by the coordinated expression levels of CHIT1 and ACTA2 in pancreatic cancer tumor tissues. These bioinformatics findings provide new clues to further explore the crosstalk between bM2-like TAMs and stromal cells in PDAC progression and therapy.

Taken together, our study used SLC12A5 and ENPP2 to define a bM2-like TAMs subcluster in PDAC by integrating single-cell sequencing and bulk sequencing, and revealed that the bM2-like TAMs might activate T cells through ALCAM-CD6 interaction and regulate the α-SMA+/α-SMA-ratio by secreting TGF-β (Figure 5E). Our findings may provide clinical markers for PDAC prognosis prediction and PDAC immunotherapy by converting pro-tumor TAMs into anti-tumor TAMs. This study has several limitations. First, despite our detailed analysis of the characterization and function of bM2-like TAMs at the single-cell transcriptome level, these bioinformatics results still require further experimental validation. Second, the key mechanisms that determine the differentiation of bM2-TAMs/mM2-like TAMs are still unclear. We plan to conduct more in-depth explorations using *ex vivo* and *in vivo* models in the future. Third, the results of the TAMs in this study will also need to be validated with other scRNA-seq data as the available datasets are updated. Limited by the public dataset, there may be unknown confounding factors interfering with prognosis-related conclusions. When moving to the bulk data (where the proportion of TAMs is likely relatively low), identified differences in any gene might be attributed to other type and might not necessarily one of our TAMs.

## 5 Conclusion

In conclusion, we identified a novel anti-tumor subcluster of M2-like TAMs with high expression of SLC12A5 and ENPP2 as bM2-like TAMs. We further demonstrated that high levels of bM2-like TAMs may suppress PDAC progression and contribute to a favorable prognosis by enhancing T-cell activation and remodeling intratumoral stroma formation. Our findings provide new insights into the highly heterogeneous nature of the TME and provide new ideas for the application of immunotherapy in PDAC.

## Data Availability

The original contributions presented in the study are included in the article/Supplementary Material, further inquiries can be directed to the corresponding authors.

## Glossary

PDAC: Pancreatic Ductal Adenocarcinoma
TME: Tumor Microenvironment
TAMs: Tumor Associated Macrophages
SLC12A5: Solute Carrier Family 12 Member 5
ENPP2: Ectonucleotide Pyrophosphatase/Phosphodiesterase 2
C1QC: Complement C1q C Chain
CXCL9: C-X-C Motif Chemokine Ligand 9
ALCAM: Activated Leukocyte Cell Adhesion Molecule
αSMA/ACTA2: Actin Alpha 2, Smooth Muscle
SPP1: Secreted Phosphoprotein 1
MIF: Macrophage Migration Inhibitory Factor
PAAD: Pancreatic Adenocarcinoma
KRT18: Keratin 18
EPCAM: Epithelial Cell Adhesion Molecule
PECAM1: Platelet And Endothelial Cell Adhesion Molecule 1
COL3A1: Collagen Type III Alpha 1 Chain
PTPRC: Protein Tyrosine Phosphatase Receptor Type C
MKI67: Marker Of Proliferation Ki-67
ADM: Acinar Ductal Metaplasia
C1QB: Complement C1q B Chain
C1QA: Complement C1q A Chain
HLA-DPA1: Major Histocompatibility Complex, Class II, DP Alpha 1
HLA-DQA1: Major Histocompatibility Complex, Class II, DQ Alpha 1
HLA-DPB1: Major Histocompatibility Complex, Class II, DP Beta 1
ROS: Reactive Oxygen Species
NO: Nitric Oxide
VEGFA: Vascular Endothelial Growth Factor A
PIGF: Phosphatidylinositol Glycan Anchor Biosynthesis Class F
TNF: Tumor Necrosis Factor
IL-17: Interleukin 17
HIF-1: Hypoxia Inducible Factor 1
CADM: Cell Adhesion Molecule
HLA-DR: Major Histocompatibility Complex, Class II, DR
PSCs: Pancreatic Stellate Cells
CAFs: Cancer-associated Fibroblasts
TGFβ: Transforming Growth Factor Beta
SMAD3: SMAD Family Member 3
SMAD5: SMAD Family Member 5
ECM: Extracellular Matrix
CHIT1: Chitinase 1
CRC: Colorectal Cancer
