# Supplementary material for: Single-cell transcriptome analysis identifies a novel tumor-associated macrophage subtype predicting better prognosis in pancreatic ductal adenocarcinoma
Source: Front Cell Dev Biol. 2024 Oct 23;12:1466767. doi: 10.3389/fcell.2024.1466767 (PMC11537994; doi:10.3389/fcell.2024.1466767)
Supplement: Supplementary file 1 [file DataSheet1.docx]

Supplementary Material

# Supplementary Figures


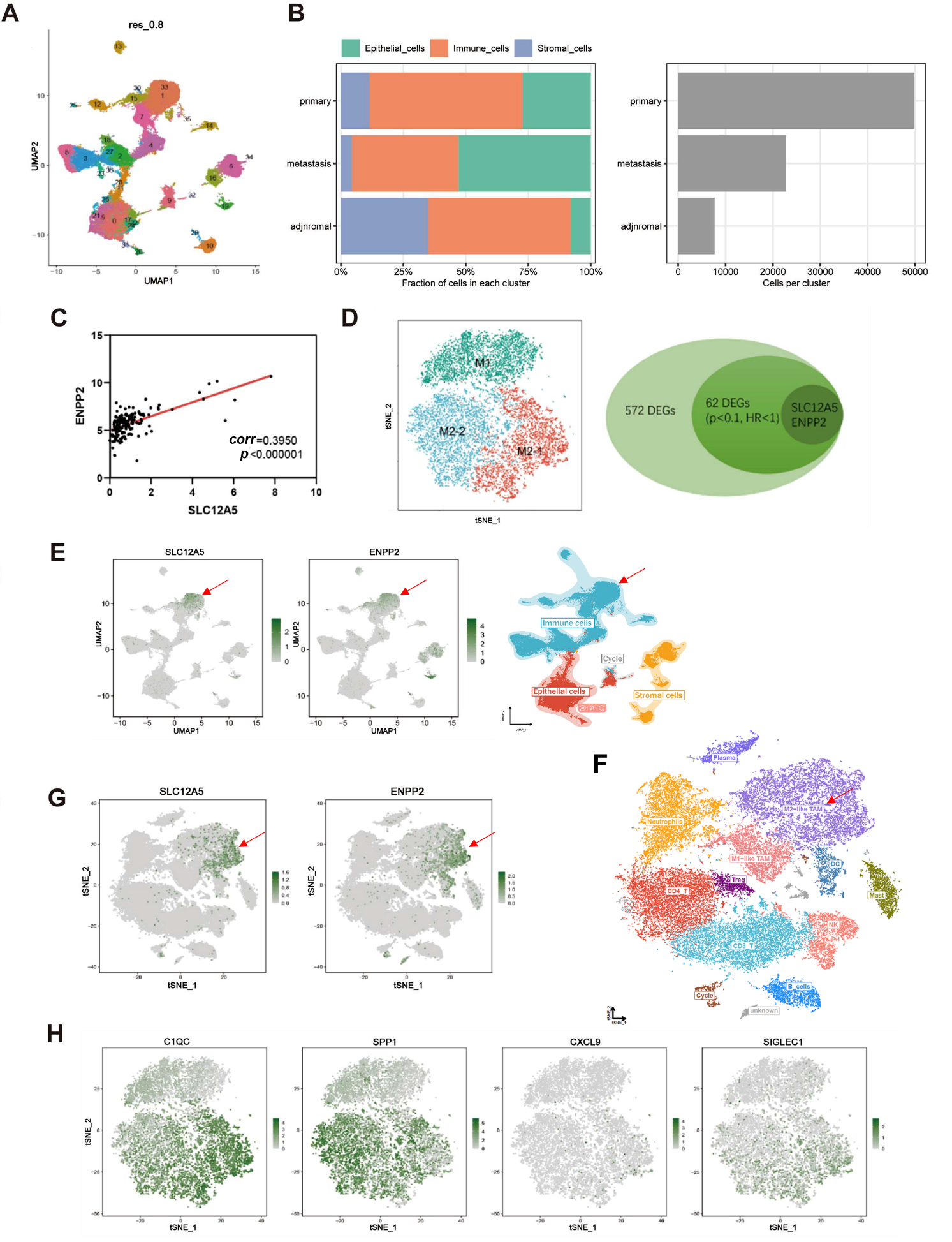


**
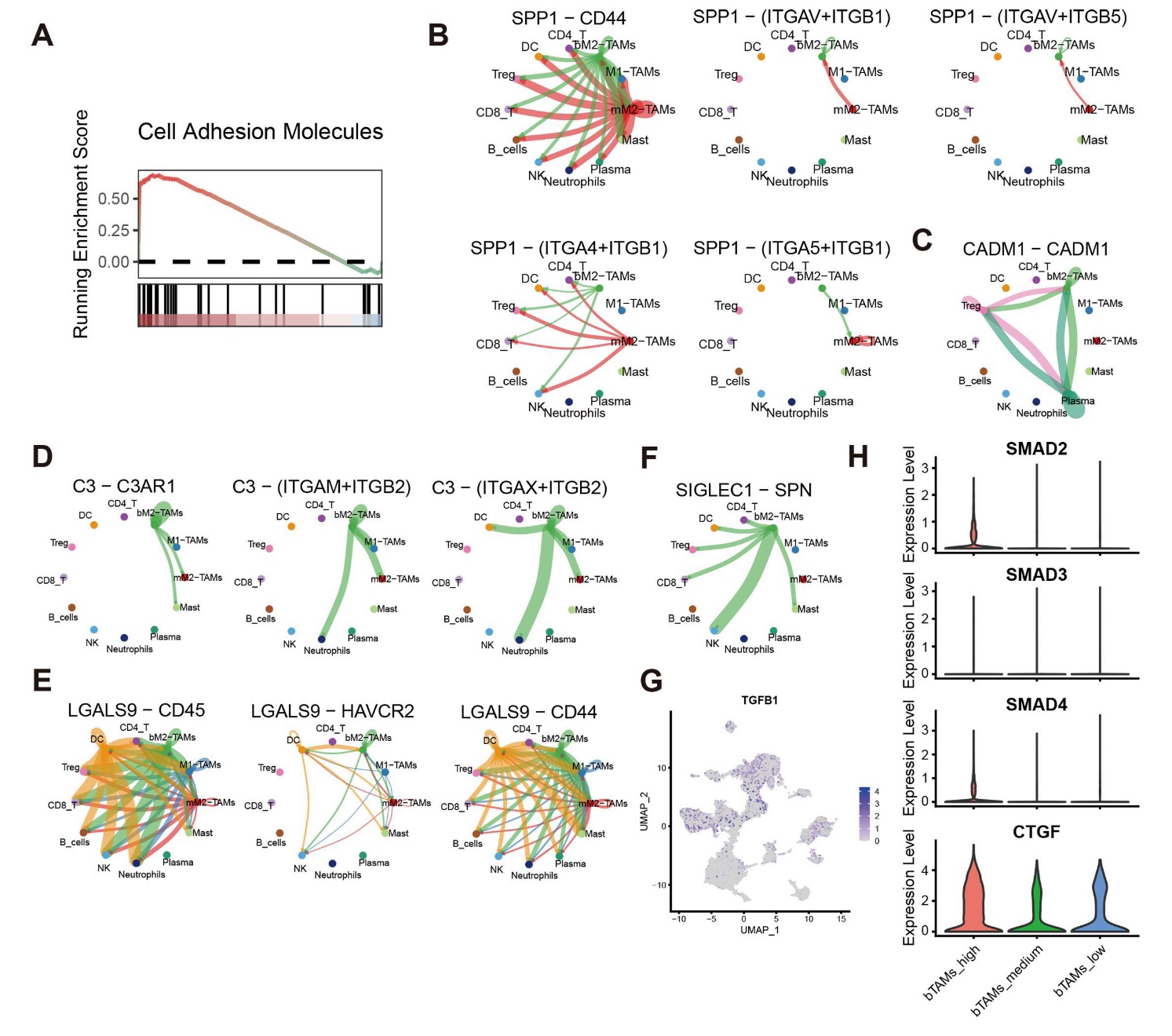
Supplementary Figure 1.** Data preprocessing and identification of TAMs subclusters. Related to Figure 1-2. **(A)** Umap visualization of the 37 clusters of PAAD scRNA data identified by FindClusters function at resolution of 0.8. **(B)** Cell fractions (left) and counts of primary tumor, metastasis and adjacent normal tissue. **(C)** Linear regression of SCL12A5 and ENPP2 in a large RNA-seq cohort of 172 PAAD patients. **(D)** TAMs were revealed to be composed of three subclusters (M1, M2-1 and M2-2) and found two prognosis-associated genes. **(E)** SLC12A5 and ENPP2 expression profiles in all celltypes. **(F)** t-SNE visualization of immune cells. **(G)** Distribution of SLC12A5 and ENPP2 in immune cells. **(H)** Distribution of C1QC, SPP1, CXCL9 and SIGLEC1 (also known as CD169) in TAMs.

**Supplementary Figure 2.** Downstream biofunction of bM2-like TAMs. Related to Figure 3-5. **(A)** GSEA analysis of the DEGs between bM2-like TAMs and mM2-like TAMs revealed the upregulation of cell adhesion molecules in bM2-like TAMs. CellChat analysis results of SPP1 pathway. **(B)** CADM pathway **(C)** complement pathway **(D)** galectin pathway **(E)** and SN pathway **(F)** were visualized by circle plot. **(G)** Distribution of TGFB1 in all cell types. **(H)** Expression of downstream effectors of TGFβ in patients with high, medium or low bM2-like TAMs fraction **
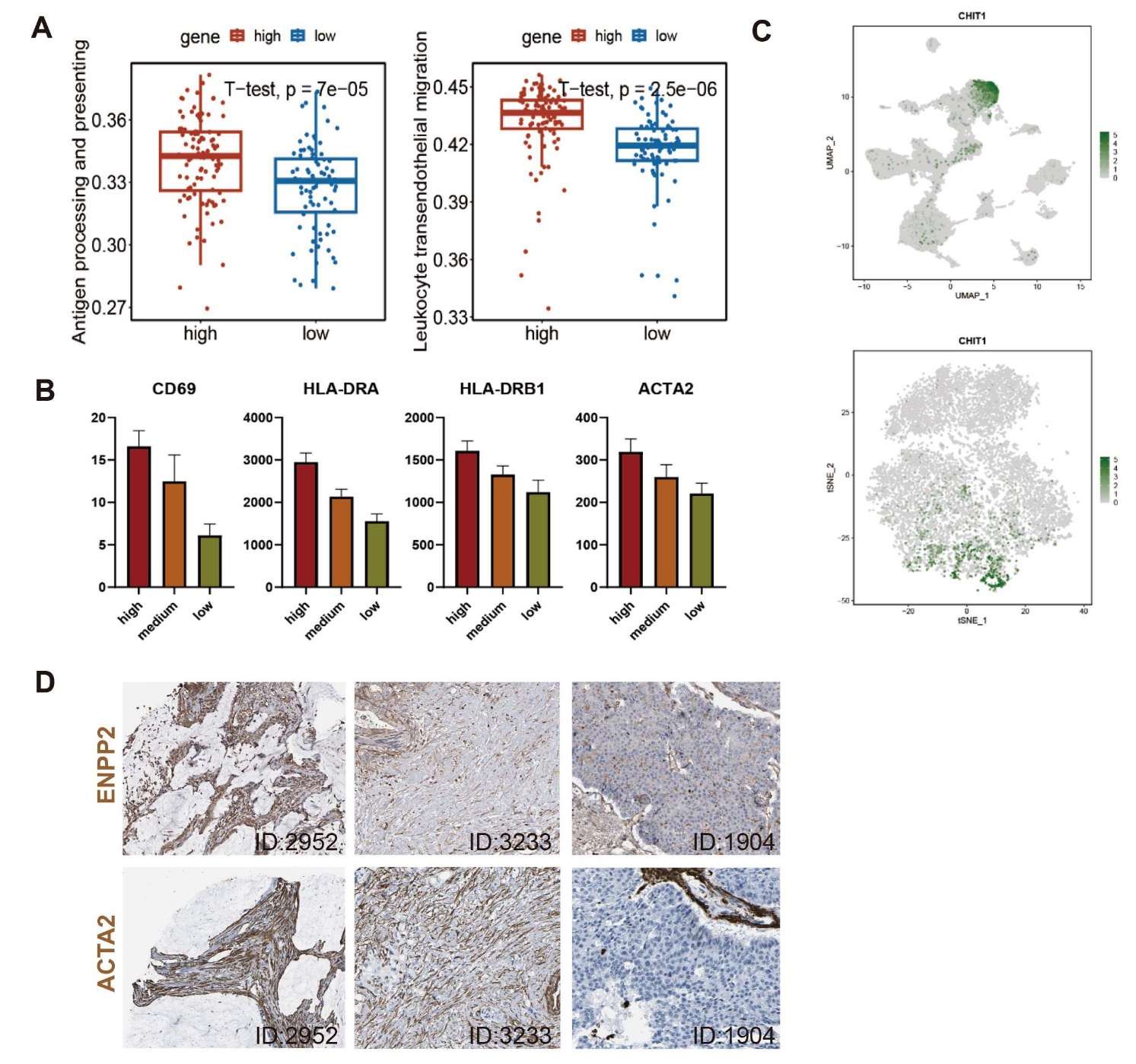
**(n(bTAMs_high) = 13, n(bTAMs_medium) = 13, n(bTAMs_low) = 14).

**Supplementary Figure 3.** Validation of the immune regulation function of bM2-like TAMs. Related to Figure 6. **(A)** ssGSEA analysis of bulk RNA-seq from TCGA dataset revealed upregulation of antigen processing and presenting pathway and leukocyte transendothelial migration pathway in patients with higher co-expression of SLC12A5 and ENPP2, p value = 7*10-5 and 2.5*10-6, respectively. **(B)** Expression level of T cell activation markers (CD69, HLA-DRA, HLA-DRB1) and αSMA+ myofibroblasts markers in TCGA PAAD cohort, grouped by the fraction of bM2-like TAMs (n(bTAMs_high) = 60, n(bTAMs_medium) = 58, n(bTAMs_low) = 60). **(C)** Distribution of CHIT1 in all cell types (top) and TAMs (bottom). **(D)** Protein expression of ENPP2 and ACTA2 in PAAD tissue, patient IDs in HPA were 2952 (left), 3233 (middle) and 1904 (right).
